# High physical activity and ovarian reserve: a prospective study of normo-ovulatory professional athletes

**DOI:** 10.1186/s13048-022-01040-x

**Published:** 2022-09-16

**Authors:** Netanella Miller, Yael Pasternak, Einat Haikin Herzberger, Hadar Gluska, Chen Dorenstein, Roni Rahav, Rina Hemi, Nahid Zada, Amir Wiser

**Affiliations:** 1 IVF Unit, Department of Obstetrics and Gynecology, Meir Medical Center, 59 Tshernichovsky St., Kfar Saba, Israel; 2 Sackler School of Medicine, Tel Aviv University, Tel Aviv-Yafo, Israel; 3 Laniado Hospital, Netanyia, Israel; 4 The Adelson School of Medicine, Ariel University, Ariel, Israel; 5 Division of Endocrinology, Diabetes and Metabolism, Sheba Medical Center, Ramat Gan, Israel

**Keywords:** Anti-Mullerian hormone, Antral follicle count, Athletes, Follicle stimulating hormone, High physical activity, Ovarian reserve

## Abstract

**Background:**

This study investigated whether high physical activity affects ovarian reserve in normo-ovulatory, reproductive-age women.

**Methods:**

This prospective, observational study compared 31 professional female athletes, with 31 women who did not engage in physical activity. It was conducted 2017–2020 in a tertiary medical center. Normo-ovulatory, professional athletes, ages 20–35 years were recruited from The Wingate Institute—the Israeli National Institute for Sport Excellence. They had high International Physical Activity Questionnaire (IPAQ) scores. Non-physically active women, matched by age and body mass index, were recruited from hospital staff. Women were evaluated for ovarian reserve markers on day 2–5 of the menstrual cycle, including follicular stimulating hormone, antral follicle count, anti-Mullerian hormone and Inhibin B.

**Results:**

The average age of the high physical activity group was 29.9 ± 4.2 years and the nonactive group 31.6 ± 4.2 years (*p* = 0.062). Body mass index of both groups were similar (22.5 ± 5.0 vs. 21.4 ± 2.5, respectively; *p* = 0.1). No differences were observed with respect to follicle stimulating hormone (*p* = 0.12) and anti-Mullerian hormone (*p* = 0.16). A trend towards higher total antral follicle count in the high physical activity group vs. the non-active group (34.5 ± 12.9 vs. 28.1 ± 15.2, *p* = 0.08) and lower Inhibin B (68.1 ± 36.8 vs. 89.4 ± 46.1, *p* = 0.05). Menarche age correlated with anti-Mullerian hormone (r = 0.387, *p* = 0.003), as did total antral follicle count (r = 0.368, *p* = 0.004). IPAQ scores and basal follicle stimulating hormone levels were negatively correlated (r = − 0.292, *p* = 0.005).

**Conclusions:**

Athletic, normo-ovulatory women have ovarian reserves that are at least as good as those of the general population. As this is the first study examining this issue, it could cautiously reassure women engaged in high physical activity regarding ovarian reserve.

## Background

There is significant evidence that physical activity has health benefits for the general population [1]. However, information regarding the effect of physical activity on the reproductive system, especially when intensive exercise is involved is lacking [2, 3].

Various mechanisms affect the reproductive system of a woman involved in high physical activity (HPA). It is well-established that the hypothalamic-pituitary-ovarian axis might be disrupted due to excessive exercise. The hormonal profile of athletes is frequently characterized by suppression of hypothalamic pulsatile release of GnRH. This results in limited pituitary secretion of luteinizing hormone (LH) and follicle-stimulating hormone (FSH), which reduces ovarian stimulation and estradiol production. With these changes, the clinical presentation is often delayed menarche, primary or secondary amenorrhea, or oligomenorrhea [4–7].

While the effect of HPA on the hypothalamic-pituitary-ovarian axis is well understood, information is still inadequate regarding the direct influence of HPA on the ovarian reserve. Ovarian reserve is defined by the functional potential of the ovary and reflects the number and quality of oocytes [8]. It is usually measured by antral follicle count (AFC) [9–11], basal follicle stimulating hormone (FSH) levels, inhibin B [12] and anti-Mullerian hormone (AMH) [13, 14].

Data show that regular moderate physical activity positively influences ovarian reserve and assisted reproductive treatment (ART) outcomes in overweight and obese women [15–18]. A few studies also suggested that moderate physical activity improves ovarian reserve in normal weight, reproductive age women [19, 20]. However, it is not yet known whether HPA influences the ovarian reserve of normo-ovulatory, reproductive-age women.

The purpose of this prospective cohort study was to examine the effect of HPA on ovarian reserve, as assessed by biological and sonographic markers, in normal weight, normo-ovulatory reproductive age women, with no alterations in the hypothalamic-pituitary-ovarian axis. The study focused on professional athletes as a reference group.

## Results

A total of 62 women were recruited for the study, of whom 31 were athletes (the HPA group), and 31 women who did not engage in physical activity (the control group).

### Basic characteristics

Table 1 shows the demographic and clinical characteristics of both groups. The ages of the women in the HPA and non-active groups were similar (29.9 ± 4.2 vs., 31.6 ± 4.2, *p* = 0.062). The average BMI in both groups was in the normal range (22.5 ± 5.0 vs. 21.4 ± 2.5, *p* = 0.1). More women in the HPA group were nulliparous (97%) as compared to the non-active group (80.6%, *p* = 0.001). Age at menarche was similar between the HPA and non-active groups (13.4 ± 2.1 vs. 12.7 ± 1.2, respectively; *p* = 0.1). The IPAQ score was higher in the HPA group compared to the non-active group (12,245.9 ± 9950.7 vs. 1462.7 ± 1137.4, *p* < 0.001). All the women recruited to the study were healthy, with no chronic diseases and no history of surgery that might have compromised ovarian reserves.

**Table 1:** Demographic and clinical characteristics of High Physical Activity and Non-active Groups

Characteristic (average ± SD)	HPA (***n*** = 31)	Non-active (***n*** = 31)	P-value
Age, years	29.9 ± 4.2	31.6 ± 2.2	0.062
Body mass index, kg/m^2^	22.5 ± 5.0	21.4 ± 2.5	0.1
Nulliparity, n (%)	30 (97)	25 (80.6)	**0.001**
Smoker, n (%)	4 (13)	7 (22)	0.412
Menarche age, years	13.4 ± 2.1	12.7 ± 1.2	0.1
IPAQ score (MET-min/wk)	12,245.9 ± 9950.7	1462.7 ± 1137.4	**< 0.001**

*HPA* High Physical Activity, *IPAQ* International Physical Activity Questionnaire, *MET-min/wk* Metabolic Equivalent Task minutes per week

Table 2 demonstrates the different types of sports that the HPA group participated in. Running and Strength and conditioning workout were the most popular, each practiced by slightly more than 25% of the HPA group.

**Table 2:** Distribution of sport type in the High Physical Activity group

Sport	Number	%
Running	8	25.8
Strength and conditioning workout	8	25.8
Weightlifting	4	12.9
Other	3	9.7
Basketball	2	6.5
Kickboxing	2	6.5
Swimming	2	6.5
Wrestling	2	6.5

### Ovarian reserve in HPA vs. control groups

Biochemical markers and sonographic indicators of ovarian reserve are described in Table 3. No significant difference was noticed in AMH and FSH measurements between the HPA and the non-active groups (*p* = 0.12 and *p* = 0.16, respectively). The basal FSH level in both groups was < 10 pg/mmol (estradiol levels were < 200 pmol/l), which is considered a normal ovarian reserve [12]. AMH levels for both groups were also normal and even in the range of high ovarian reserve based on AMH nomograms [21, 22].

**Table 3:** Biochemical markers and sonographic indicators of ovarian reserve of HPA and Non-active Groups

Basic characteristics (average ± SD)	HPA (*n* = 31)	Non-Active (*n* = 31)	*P*-value
Basal FSH (pg/mmol)	5.6 ± 2.6	6.5 ± 1.8	0.12
Inhibin B (pg/ml)	68.1 ± 36.8	89.4 ± 46.1	0.05
Anti-Mullerian hormone (ng/ml)	6.26 ± 3.3	4.96 ± 3.7	0.16
Antral follicular count (n)	34.5 ± 12.9	28.1 ± 15.2	0.08

*HPA* High Physical Activity, *FSH* Follicle Stimulating Hormone

A trend towards higher total AFC was noticed in the HPA group compared to the non-active group (34.5 ± 12.9 vs. 28.1 ± 15.2, respectively; *p* = 0.08), and nearly significant lower Inhibin B level was found in the HPA group (68.1 ± 36.8 vs. 89.4 ± 46.1, respectively; *p* = 0.05).

### Correlations between demographic and clinical characteristics and ovarian reserve

The main results of the correlations between demographic and clinical characteristics and ovarian reserve measurements for the entire cohort are presented in Table 4. Age at menarche was positively correlated with AMH (r = 0. 387, *p* = 0.003), as well as with total AFC (r = 0.368, *p* = 0.004). A negative correlation was also found between IPAQ score and basal FSH levels (r = − 0.292. *p* = 0.005). A sub-analysis of correlation for the HPA group found negative correlations between BMI and AMH and AFC (*p* = 0.05 and *p* = 0.029, respectively). These differences were not seen in the non-active group.

**Table 4:** Correlations between demographic and clinical characteristics and ovarian reserve measurements

Variable	Age	BMI	Menarche age	IPAQ score	AMH	FSH	Inhibin B	Total AFC
Age	1	–	–	–	–	–	–	–
BMI	−0.003	1	–	–	–	–	–	–
Menarche age	−0.190	−.281^a^	1	–	–	–	–	–
IPAQ score	−0.164	0.086	0.053	1	–	–	–	–
AMH	−0.229	−0.171	0. 387^b^	0.187	1	–	–	–
FSH	0.090	−0.058	0.087	−0.292^a^	−0.254	1	–	–
Inhibin B	−0.043	−0.256	0.027	−0.020	0.167	0.225	1	–
Total AFC	−0.260^a^	−0.119	0.368^b^	0.180	.765^b^	−0.207	0.121	1

*IPAQ* International Physical Activity Questionnaire, AMH: Anti-Mullerian Hormone, *FSH* Follicle Stimulating Hormone, *AFC* Antral Follicular Count

^a^Significant at the 0.05 level (two-tailed). ^b^Significant at the 0.01 level (two-tailed)

Multivariable logistic regression analysis for AMH showed that inhibin B and AFC were significant variables (*p* = 0.014 and *p* = 0.001, respectively). FSH showed a negative trend (*p* = 0.057). IPAQ score and HPA were not significant (*p* = 0.682 and *p* = 0.810, respectively). Multivariable logistic regression analysis for AFC showed that only AMH was a significant variable (*p* = 0.001).

## Discussion

The main findings of this study suggest there are no significant differences in ovarian reserve measurements between normo-ovulatory women based on physical activity. AMH levels and basal FSH levels were similar between the HPA and the non-active groups. However, the trend towards higher total AFC in the HPA compared to the non-active group, and the negative correlation between IPAQ score and basal FSH levels may indicate more favorable ovarian reserves in the HPA group.

Most exercise-associated reproductive abnormalities are derived from dysfunction at the hypothalamic level. The hormonal profile of athletes, especially those who are involved in sports that emphasize low weight, is typically characterized by hypoestrogenism resulting from disruption of the hypothalamic-pituitary-ovarian axis. A prolonged follicular phase or absence of a critical LH or estradiol surge mid-cycle, results in the mild or intermittent suppression of menstrual cycles observed in these women [4–7]. It is important to note that exercise coupled with caloric restriction was shown to effect LH suppression, while exercise alone does not affect LH pulsatility [23]. By including only women with normal BMI in this study and excluding those with eating disorders, we excluded athletes with hypothalamic disfunction. Therefore, we were able to focus solely on the effect of physical activity on ovarian reserve, as few studies have done previously.

A possible explanation for the few favorable results regarding ovarian reserve in the HPA group is that physical activity enhances blood flow to crucial organs. There is sufficient information regarding blood flow to essential organs during intense physical activity [24], whereas there is no information regarding blood flow to human ovaries during exercise. However, one study showed that the blood flow through both ovarian arteries in mares was greater in both partially and fully exercised groups in the days leading up to ovulation, compared to controls [25].

We found nearly significant lower levels of Inhibin B in the HPA group. Yet, it is uncertain whether this has clinical implications that indicate lower ovarian reserves in this group. The average values of Inhibin B were within the normal range for age (10–200 pg/ml) in both groups [22]. Moreover, the cascade of events reflecting decreased ovarian reserve would include declining levels of inhibin B that in turn would be reflected by rising FSH levels. This finding was not seen in our study and as mentioned above, FSH levels were negatively correlated with IPAQ score and AFC was positively correlated. Lastly, it has been shown that in the general population, inhibin B was less predictive of menopause than was AMH [22, 26].

An interesting finding in this study was the positive correlation between late menarche and AMH levels and total AFC. Similar findings were seen in a few previous studies. Weghofer et al. found that women who experienced menarche at a younger age were more likely to have abnormal, age-specific decreased functional ovarian reserve [27]. Another study showed that AMH concentrations were significantly lower among women who reached menarche at a younger age [28]. Another study, that included 2030 healthy women, also suggested a positive association between AMH concentrations and age at menarche, although the results were only marginally significant [29]. The assumption regarding these cohorts was similar: earlier menarche might lead to earlier follicular depletion. The theory is based on the evidence that follicular recruitment peaks during puberty and declines thereafter [30].

It is important to mention that we did not find any correlations regarding BMI and ovarian reserve measurements for the entire cohort. This might be because both study groups were matched by BMI, and that the average BMI of both groups was in the normal range (18.5–24.9) [31].

Previous studies reported contradictory results regarding BMI and ovarian reserve. Some presented that high BMI is an independent factor for significantly lower AMH levels [32–34], while others found no difference in AMH levels amongst women with high compared to normal BMI [28, 29, 35–39]. Low AMH reflects decreased follicular function, which could be a result of the inflammatory markers in the ovaries, secreted in cases of obesity [40, 41]. Nevertheless, among women with obesity and anovulation, it is common to find more small antral follicles that secrete AMH [42].

An interesting finding was that both the HPA and the non-active groups had comparatively high levels of AMH based on a known nomogram for AMH [43]. Possible explanations are that the cohort included young women, ages 20–35 years. Younger women are known to have higher AMH levels. Moreover, the age-related nomograms of serum anti-Müllerian hormone levels are based on infertile women and not on the general population. Fertile women may have higher AMH levels compared to infertile women.

When analyzing only the HPA group, we found negative correlations between BMI and AMH and AFC. It is possible that the relatively higher BMI in this group neutralized the potentially positive effect of higher blood flow, due to increased levels of inflammatory markers. Further studies should be conducted to evaluate this effect.

Our study has several strengths. It is the first prospective study aimed to examine the effect of HPA on ovarian reserves in normo-ovulatory women. The fact that we used professional athletes as the reference for the HPA group, strengthens our analysis. Another strength is that all ultrasound measurements were taken by the same skilled sonographer, which eliminates inter-observer variability. In addition, physical activity levels of the participants were evaluated and categorized by IPAQ, a standard, reliable, validated international tool.

A limitation of this study relates to its relatively small sample size. Moreover, there were more nulliparous women in the HPA group. It is possible that parity may affect ovarian reserve, but that could not be evaluated precisely in this study. Another limitation is the diversity of sport types in the HPA group. Ideally, we would have designed a study that explored the influence of each type of sport on ovarian reserves. We were not able to recruit enough women from each sport to obtain sufficient statistical power. Further studies with larger cohorts are needed to evaluate this question.

## Conclusions

This study presents data showing that normo-ovulatory women who engage in HPA have an ovarian reserve that is at least as good as that of the general population. Since this is the first study examining this question, it could cautiously provide women engaged in HPA with reassurance regarding their ovarian reserves. More studies are required to reinforce this conclusion.

## Methods

### Study design

This prospective, observational study compared 31 professional female athletes who were engaged in high physical activity (HPA) for at least 3 years before study recruitment and had high International Physical Activity Questionnaire (IPAQ) scores, with 31 women who did not engage in physical activity. The study was conducted at a tertiary medical center from 2017 to 2020.

### Study population

Physically active, normo-ovulatory women (*n* = 31), ages 20–35 years were recruited from The Wingate Institute—the Israeli National Institute for Sport Excellence. Non-physically active women (*n* = 31), matched by age and BMI to the HPA group, were recruited from the hospital staff.

Inclusion criteria were women ages 18 to 35 years, with BMI 18 to 30 kg/m^2^. Exclusion criteria included women who used contraceptives, women with a known prior diagnosis of polycystic ovary syndrome, endometriosis or premature ovarian failure.

Participants who met the eligibility criteria were evaluated for ovarian reserve markers on day 2–5 of the menstrual cycle, including follicular stimulating hormone (FSH), anti-Mullerian hormone (AMH), Inhibin B and antral follicle count (AFC).

### Measures

#### Questionnaires

Demographic and fertility information were obtained on the day of assessment. Physical activity was assessed using the IPAQ. This questionnaire was developed by the World Health Organization in 1998 and is used to obtain internationally comparable estimates of physical activity (“www.ipaq.ki.se”) [44]. The data in the questionnaire are computed by weighting each type of activity by its energy requirements, to yield a score that is converted to Metabolic Equivalent Task minutes per week (MET-min/wk). METs are multiples of the resting metabolic rate, and a MET-minute is the MET score of an activity multiplied by minutes performed. HPA is defined by either vigorous-intensity activity on at least 3 days and accumulating at least 1500 MET-min/wk. or 7 or more days of any combination of walking, moderate-intensity or vigorous intensity activities achieving a minimum of 3000 MET-minutes/week. The selected MET values were derived from work undertaken during the IPAQ Reliability Study [45].

#### Blood markers

For evaluation of FSH, AMH and Inhibin B levels, we collected 10–15 ml of blood. Serum AMH and inhibin B concentrations were measured using enzyme-linked immunosorbent assays (AMH Gen II ELISA and Inhibin B GEN II ELISA; Beckman Coulter, Prague, Czech Republic). AMH intra- and inter-assay coefficients of variations were > 10.3 and 10.8%, respectively. Inhibin B intra- and inter-assay coefficients of variations were 5.6 and 6.6%, respectively. Basal FSH was taken between days 2–5 of the menstrual cycle.

#### Sonographic marker

Antral follicle count was measured on the assessment day by transvaginal ultrasound. The same experienced ultrasound technician conducted the exam for all participants. AFC was calculated as the number of 2 to 9 mm follicles seen in both ovaries. These measures were compared between the HPA and the non-active groups.

### Statistical analysis

Data analysis was performed using SPSS 24.0 package for windows (IBM Corp., Armonk, NY). Discrete variables are presented as numbers and percentages and continuous variables as means and standard deviations (SD). We calculated *p*-values using t-test or χ^2^ for continuous and categorical variables, respectively. Pearson’s coefficients were calculated between relevant variables. Multivariate regression was conducted to evaluate variables affecting AMH and AFC. For all tests, a p-value < 0.05 was considered significant.

Regarding sample size, assuming that the average mean AMH level for the non-active group is 4.7 ng/ml, the study would require a sample size of 30 in each group to achieve a power of 80% and a two-sided significance level of 5%, to detect a difference in means between the non-active and the HPA groups of − 2.2 units.

## Data Availability

The datasets used and analyzed in the current study are available from the corresponding author on reasonable request.

## Abbreviations

AFC: Antral Follicle Count
AMH: Anti-Mullerian Hormone
FSH: Follicle Stimulating Hormone
IPAQ: International Physical Activity Questionnaire
MET-min/wk.: Metabolic Equivalent Task minutes per week
